# Comparison of MRI imaging features to differentiate degenerating fibroids from uterine leiomyosarcomas

**DOI:** 10.1177/20363613251327080

**Published:** 2025-04-11

**Authors:** William W Loughborough, Andrea G Rockall, Tanja T Gagliardi, Laura Satchwell, Emily Greenlay, Piers Osborne, Nishat Bharwani, Thomas Ind, Ayoma Attygalle, Dione Lother, Georgina Hopkinson, Robin Jones, Charlotte Benson, Aisha Miah, Aslam Sohaib, Christina Messiou

**Affiliations:** 1 The Royal Marsden Hospital NHS foundation trust, London UK; 2 The Royal United Hospitals Bath NHS foundation trust, Bath UK; 3 8946 Imperial College Healthcare Trust, London, UK; 4 The Institute of Cancer Research, London, UK

**Keywords:** Magnetic resonance imaging, radiology, uterine leiomyosarcoma, rare cancer types, rare tumour research

## Abstract

**Objectives:** The aim of this study was to construct a diagnostic model from MRI features to distinguish complex leiomyomas/degenerating fibroids (DF) from leiomyosarcoma (LMS). **Methods:** A retrospective case-controlled study was performed comparing MRI features of patients with pathologically proven DF or LMS. MRI in 42 patients with DF (control group) and 46 with LMS (study group) was used to generate a diagnostic model. Imaging features reported in the literature to distinguish these two entities were scored for each uterine mass by two radiologists unaware of the histological diagnosis. Inter observer variation and univariate analysis was undertaken. Imaging characteristics identified on univariate analysis were used to build a multi-variable diagnostic model and sensitivity and specificity of this model calculated. **Results:** Taking the features identified on the univariate analysis, the final diagnostic model was based on AP length (*p* = .053), intermediate T2 signal (IT2), volume (*p* = .002), and nodular border (*p* = .001). When the model was implemented back into the training dataset it demonstrated a sensitivity of 70.7%, and a specificity of 76.2%. The sensitivity and specificity of radiologist suspicion score was 74.7% and 70.4%. In addition, morphological features showed only poor or moderate inter observer agreement at best. **Conclusions:** Morphological MRI imaging features alone are not sufficient to obviate the need for pathological confirmation prior to non-surgical management of complex uterine mass lesions. **Trial registration:** IRAS project ID 251778 Protocol number: CCR 4992 REC reference 19/YH/0134 Date of HRA approval: 29.4.19.

## Advances in knowledge

Morphological features of uterine masses on MRI are not sufficient to distinguish leiomyosarcoma from degenerating fibroids.

## Introduction

Uterine fibroids (leiomyomas) are the commonest benign uterine tumour affecting 20%–40% women of reproductive age.^1–3^ Leiomyosarcoma (LMS) is a rare but highly aggressive myometrial malignancy with an incidence highest in the 50-54 year age group.^4^ Symptomatic fibroids can be managed by a range of conservative, minimally invasive or surgical management options. However, LMS requires management at a high-volume sarcoma and gynaecology oncology centre and in the absence of metastatic disease usually involves complete surgical resection with total abdominal hysterectomy and bilateral salpingo-oophorectomy. A systematic analysis by the US Food and Drug Administration estimated that the prevalence of unsuspected uterine leiomyosarcoma in women undergoing hysterectomy or myomectomy for presumed benign leiomyoma is 1 in 498 women.^5^ A retrospective series of women undergoing morcellation for suspected fibroids, but with undiagnosed uterine LMS, reported worse outcomes.^6^ Uterine artery embolization followed by curettage can also result in disease dissemination.^7^ Accurate pre-operative diagnosis is therefore essential to ensure safe and effective management. The myometrial location of indeterminate masses makes biopsy challenging,^8^ and therefore imaging is of increasing importance.

Degenerating fibroids (DFs) can have a heterogeneous appearance on MRI mimicking LMS.^9^ However, there are very few published studies on the effectiveness of imaging in distinguishing DFs from LMS. Cohorts prior to this study are limited to fewer than 20 cases of LMS and often include a range of sarcoma subtypes other than LMS.^10–17^ Other studies propose age as an important risk factor as during resection for a presumed fibroid, patients aged 75–79 years have a 1 in 98 chance of having an unexpected LMS resected, compared with a 1 in 547 risk in women aged 25–29.^18–20^

The aim of this study was to construct a diagnostic model of MRI features to distinguish DF from LMS on a training set which could then be applied to an independent validation data set. The secondary objective was to establish inter-observer variability for each MRI feature.

## Material and Methods

This was a retrospective 1:1 case-control study. The study protocol was approved by the local committee for clinical research and by the NHS (National Health Service) Health Research Authority Research Ethics Committee (Yorkshire and The Humber-South Yorkshire Research Ethics Committee 19/YH/0134 and patient consent was waived as the study used fully anonymized retrospective data.

### Inclusion and exclusion criteria

Inclusion criteria:

• Histologically proven DF or LMS.
• Minimum pre-treatment MRI dataset - axial T1W and axial T2W MRI pelvis with either sagittal and/or coronal T2W MRI pelvis.
• Complex DF defined on MRI by; >20% high T2 signal and/or two or more areas of intermediate T2 signal.

Exclusion criteria:

• Lack of histological confirmation
• Non-leiomyosarcoma uterine sarcoma including (smooth muscle tumours of uncertain malignant potential) STUMP
• Incomplete MRI dataset
• Fibroids not meeting MRI inclusion criteria.

### Subjects

Patient’s age, source of histology and lactate dehydrogenase (LDH) level^11,13^ were recorded.

DF cohort: A list of consecutive patients with histologically proven benign fibroids either at hysterectomy or myomectomy were identified from a large gynaecology unit at Imperial College Healthcare Trust and inclusion and exclusion criteria were applied.

LMS cohort: Consecutive patients with histologically proven uterine LMS were identified from the prospectively maintained database of the soft tissue sarcoma unit at The Royal Marsden Hospital, (2008-2018). Inclusion and exclusion criteria were applied.

All cases had been reviewed by specialist pathologists at the tertiary referral centres.

All anonymised MRI scans were randomly assigned on the PACS (picture archiving and communication system) system.

### Image Analysis

Two gynaecology-oncology radiologists, each with >10 years’ experience (AS and TG) independently performed image scoring unaware of the histology.

The case report form (CRF) (See Supplemental) was devised following a literature review of published studies describing characteristics reported to distinguish LMS from DFs (see Supplemental for reporting lexicon).

The CRF included lesion size and location (submucosal, intramural or subserosal); 3 perpendicular measurements; lesion morphology (protrusion into the myometrium, nodular border, organ invasion, feeding/internal vessels, multifocality and volume of intermediate T2, low T2 or high T1 signal). Presence of abnormal lymph nodes, ascites and peritoneal disease were also recorded. Where post contrast and DWI sequences were available, enhancement and volume of restricting components were documented. The radiologists own level of suspicion for LMS was scored on a Likert scale^21^ (very unlikely 0; unlikely 1; unsure 3; likely 4; very likely 5).

## Data Analysis

Using the rated MRI imaging features, a scoring system was built and tested. Sensitivity and specificity thresholds of the scoring system were pre-specified at 85% and 80% respectively to decide if a validation study would be justified.

### Primary Endpoint

The sensitivity and specificity of the proposed MRI diagnostic classification model was generated by using the consensus of two radiologists.

### Secondary Endpoint

The inter-observer variability for each MRI feature, expressed as Kappa and Bland-Altman limits of agreement (LoA) between radiologists.

### Statistical Analysis

#### Sample Size

As this is a non-trial study design and all available data was used, the sample size was pragmatic and restricted to around 70 patients for the training study, with 1:1 consecutive matching between LMS and DF. If the training set met sensitivity/specificity thresholds, a separate sample would be used for the validation study.

### Construction of the Diagnostic Classification Model

#### Step 1

Initial descriptive analysis of the dataset was performed to review the distributions, missingness, and collinearity between all MRI features, by investigating summary statistics, correlation matrices, and histograms by histological subtype (LMS or DF), where applicable.

Variables were deemed to have insufficient data for further analysis if there was: a large proportion (>25%) missing or not applicable; perfect prediction and/or a correlation coefficient >0.9 with another variable; or inadequate/highly skewed subgroup numbers (e.g. 95% yes and 5% no).

For the variables that were included for further analysis (Step 2), descriptive statistics have been reported in the results by radiologist and histological subtype, where applicable.

#### Step 2

To evaluate the univariate associations with outcome (histological subtype) for all remaining potential predictor variables, simple logit models were used to test each separately. All models included radiologist as a fixed covariate to account for the data being paired. As age of patient was not an MRI feature rated by the radiologists, the association between age and outcome was assessed using a *t* test. Age was included for further analysis regardless of the *t* test significance level because of the clinical rationale that it is expected to be related to outcome.

Possible predictors were taken forward to Step 3 if the logit models demonstrated univariate associations at the *p* < .1 level.

#### Step 3

The inter-observer variability between the two radiologists for each MRI feature was tested using the following parameters and acceptable ranges:

### Discrete variables

Spearman’s rank coefficient>0.5 and <10% outside the Bland Altman 95% LoA

### Continuous variables

Pearson’s correlation coefficient>0.5 and <10% outside the Bland Altman 95% LoA

### Categorical variables

Kappa Coefficient of agreement>0.4 And/or >50% overall Agreement

For MRI features that did not fall within the above criteria, they were deemed unacceptable for consideration into the multivariable model (Step 4), as this demonstrates little or no agreement between radiologists, which implies the variable is too subjective or unreliable.

#### Step 4

All variables carried forward from the previous steps, including age, were included in the final multivariable model step, where radiologist was set as a fixed covariate and histological subtype was the outcome.

Using a manual backwards stepwise selection process, the covariate with the largest *p*-value was removed in an iterative procedure until all remaining covariates had a *p*-value of <0.1.

The remaining covariates made up the final diagnostic classification model to be tested for sensitivity and specificity.

### Sensitivity analysis

Two checks were run on the final diagnostic classification model. Firstly, it was confirmed that the coefficients were not meaningfully altered by removing the fixed covariate radiologist; and secondly, it was tested that the same model was chosen when using Stata’s automated Stepwise regression command, rather than manual selection.

### Sensitivity and specificity of the diagnostic classification model

The sensitivity, specificity, positive predictive value (PPV) and negative predictive value (NPV) were calculated for the diagnostic classification model. The ROC (receiver operator characteristic) area under the curve will also be presented as an overall measure of the performance of the model.

### Sensitivity and specificity of radiologist suspicion score as a diagnostic tool

A score of 1-3 is considered low suspicion with a predicted diagnosis of DF. A suspicion score of 4-5 is considered a high suspicion score with a predicted diagnosis of LMS. Using these cut offs sensitivity, specificity, positive predictive value (PPV) and negative predictive value (NPV) were calculated from the radiologist suspicion score. The ROC area under the curve will also be presented as an overall measure of the performance of the model.

### Sensitivity analysis

The diagnostics above were also produced using a cut-off from the suspicion score of 3 for diagnosis of LMS. Those with a score of 3-5 were considered a high suspicion score with a predicted diagnosis of LMS.

All analyses were performed using Stata version 17 (StataCorp, Texas).

## Results

### Analysis population

The mean (SD – standard deviation) age in the DF group was 54.0 years (13.8) and in the LMS group was 56.2 years (10.7). LDH was only recorded in 6 patients and has therefore not been presented. DWI, T1W high signal and contrast enhanced imaging were also excluded due to paucity of data (56%, 51% and 54% respectively missing).

The total number of readings from the original dataset was 194: 99 (51%) DFs, 94 (48%) LMSs, and one missing. Of these, 18 were excluded as they were only scored by one radiologist, which may bias the results, or if histological subtype was missing.

Therefore, the total number of readings included in the analysis was 176, which accounted for 88 pairs of images from 72 patients: 84 (48%) DFs and 92 (52%) LMSs.

For the initial analysis, each radiologist assessed 42 cases of DF and 46 of LMS.

### Descriptive statistics

As described in the methodology, variables included in Step 2 onwards are displayed in Table 1. The descriptive statistics of the level of suspicion score as a diagnostic tool, by histological subtype and radiologist are presented in Table 2.

**Table 1.** Descriptive statistics of the MRI features for the diagnostic classification model deemed to have suitable data for univariate and inter-observer analysis, by histological subtype and by radiologist.

median (IQR) or *n* (%)	Radiologist 1		Radiologist 2	
	DF *N* = 42	LMS *N* = 46	DF *N* = 42	LMS *N* = 46
Craniocaudal length (mm)	76 (38–137)	113 (87–170)	85 (40–132)	110 (86–166)
AP length (mm)	70 (43–92)	86 (71–111)	69 (40–102)	98 (78–129)
Transverse length (mm)	73 (37–110)	97 (79–126)	70 (45–115)	106 (76–130)
>1 fibroid present				
Yes	32 (76.2)	26 (56.5)	27 (64.3)	21 (45.6)
No	10 (23.8)	20 (43.5)	15 (35.7)	25 (54.4)
Location of mass in myometrium			*n* = 40	
Subserosal	15 (35.7)	16 (34.8)	14 (35.0)	7 (15.2)
Intramural	24 (57.1)	25 (54.4)	17 (42.5)	30 (65.2)
Submucosal	1 (2.4)	3 (6.5)	2 (5.0)	2 (4.4)
Other	2 (4.8)	2 (4.4)	7 (17.5)	7 (15.2)
Focal protrusion into myometrium				
Yes	13 (31.0)	33 (71.7)	7 (16.7)	13 (28.3)
No	29 (69.0)	13 (28.2)	35 (83.3)	33 (71.7)
Nodular border				
Yes	17 (40.5)	44 (95.7)	24 (57.1)	35 (76.1)
No	25 (59.5)	2 (4.3)	18 (42.9)	11 (23.9)
Feeding vessel				
Yes	33 (78.6)	43 (93.5)	12 (28.6)	15 (32.6)
No	9 (21.4)	3 (6.5)	30 (71.4)	31 (67.4)
Internal vessels				
Yes	34 (81.0)	44 (95.7)	6 (14.3)	11 (23.9)
No	8 (19.0)	2 (4.3)	36 (85.7)	35 (76.1)
IT2 solitary area				
Yes	8 (19.1)	1 (2.2)	3 (7.1)	4 (8.7)
No	34 (80.9)	45 (97.8)	39 (92.9)	42 (91.3)
IT2 subjective volume of IT2 signal within the mass			*n* = 41	*n* = 45
1%–25%	11 (26.2)	1 (2.2)	11 (26.8)	2 (4.4)
26%–50%	5 (11.9)	3 (6.5)	2 (4.9)	3 (6.7)
51%–75%	7 (16.7)	2 (4.3)	9 (22.0)	2 (4.4)
76%–100%	19 (45.2)	40 (87.0)	19 (46.3)	38 (84.4)
LT2 multifocal areas				
Yes	26 (61.9)	20 (43.5)	19 (45.2)	15 (32.6)
No	16 (38.1)	26 (56.5)	23 (54.8)	31 (67.4)
Necrosis multifocal areas				
Yes	18 (42.9)	24 (52.2)	6 (14.3)	22 (47.8)
No	24 (57.1)	22 (47.8)	36 (85.7)	24 (52.2)
Fibrotic signal on DWI				
Yes	19 (45.2)	12 (26.1)	5 (11.9)	4 (8.7)
No	23 (54.8)	34 (73.9)	37 (88.1)	42 (91.3)
Enlarged nodes				
Yes	10 (23.8)	7 (15.2)	-	4 (8.7)
No	32 (76.2)	39 (84.8)	42 (100)	42 (91.3)
Peritoneal disease				
Yes	3 (7.1)	2 (4.4)	-	3 (6.5)
No	39 (92.9)	44 (95.6)	42 (100)	43 (93.5)
Ascites				
Yes	6 (14.3)	17 (37.0)	-	3 (6.5)
No	36 (85.7)	29 (63.0)	42 (100)	43 (93.5)
Adequate images				*n* = 45
Yes	34 (81.0)	44 (95.7)	34 (81.0)	41 (91.1)
No	8 (19.0)	2 (4.3)	8 (19.0)	4 (8.9)

**Table 2.** Descriptive statistics of the Level of suspicion score as a diagnostic tool, by histological subtype and by radiologist.

median (IQR) or *n* (%)	Radiologist 1		Radiologist 2	
	DF *N* = 42	LMS *N* = 46	DF *N* = 42	LMS *N* = 46
Level of suspicion score	3 (2–3.5) *n* = 40	4 (3–4)	2 (1–4) *n* = 41	5 (4–5) *n* = 45

### Construction of the diagnostic classification model

Results from Steps 2-4 are reported in Table 3. This includes: (Step 2) the beta coefficients and associated *p*-values from the logit models to assess univariate associations with outcome; (Step 3) the correlation coefficient (Spearman’s rank or Pearson’s) for quantitative variables, or Kappa coefficient of agreement for categorical variables, to assess the inter-observer variability between the two radiologists; and (Step 4) whether each MRI feature was taken forward to the final stage of model building.

**Table 3.** Univariate associations with outcome and inter-observer agreement or correlation.

	Step 2		Step 3			Step 4
	β coef	*p*-value	Correlation or kappa coef	Mean difference (95% LoA) % Outside LoA	% Agree	Take forward
Age	-	0.250	-	-	-	Yes
Craniocaudal length (mm)	0.01	<0.001	ρ = 0.94	0.6 (−39.5, 40.6)	-	Yes
				5.7%		
AP length (mm)	0.02	<0.001	ρ = 0.88	−4.5 (−39.7, 30.8)	-	Yes
				6.8%		
Transverse length (mm)	0.01	0.001	ρ = 0.85	−2.8 (−57.9, 52.4)	-	Yes
				4.6%		
>1 fibroid (ref = yes)	0.83	0.010	κ = 0.67	-	84.1%	Yes
Location of mass in myometrium (main effects)	NA	0.452	-	-	-	No
Focal protrusion into myometrium (ref = yes)	−1.30	<0.001	κ = 0.20	-	59.1%	Yes
Nodular border (ref = yes)	−1.85	<0.001	κ = 0.27	-	68.2%	Yes
Feeding vessel (ref = yes)	−0.58	0.125	-	-	-	No
Internal vessels (ref = yes)	−1.0	0.028	κ = 0.03	-	28.4%	No
IT2 solitary area (ref = yes)	0.97	0.086	κ = −0.10	-	81.8%	Yes
IT2 subjective volume of IT2 signal within mass (main effects)	NA	<0.001	κ = 0.52	-	75.6%	Yes
LT2 multifocal areas (ref = yes)	0.64	0.038	κ = 0.51	-	75.0%	Yes
Necrosis multifocal areas (ref = yes)	−0.94	0.004	κ = 0.12	-	56.8%	Yes
Fibrotic signal on DWI (ref = yes)	0.71	0.066	κ = 0.29	-	72.7%	Yes
Enlarged nodes (ref = yes)	−0.01	0.991	-	-	-	No
Peritoneal disease (ref = yes)	−0.44	0.556	-	-	-	No
Ascites (ref = yes)	−1.42	0.006	κ = 0.10	-	75.0%	Yes
Adequate images (ref = yes)	−1.20	0.017	κ = 0.30	-	85.1%	Yes

Coef: coefficient; LoA: limits of agreement; NA: not applicable as the main effect significance value has been reported, rather than for each subgroup; ref: reference category; r = Spearman’s rank correlation coefficient; ρ: Pearson’s correlation coefficient; κ = Kappa coefficient of interrater agreement. IT2: LT2.

Out of the 19 variables reported in Table 1, 14 were taken forward to the manual backwards stepwise selection process, plus age of patient. The five that were dropped during Steps 2-4 were: location of mass in myometrium, feeding vessel, internal vessels, enlarged nodes, and peritoneal disease.

### Final diagnostic classification model

**Figure 1.**
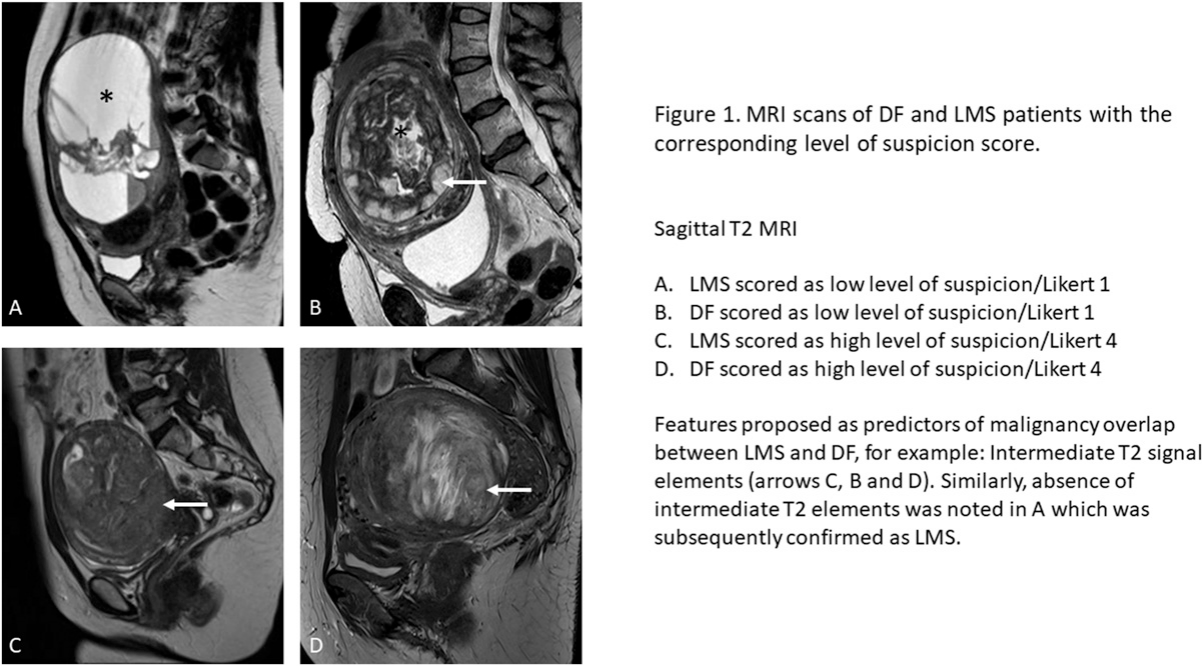
MRI scans of DF and LMS patients with the corresponding level of suspicion score.

The final diagnostic classification model is shown in Table 4. After the stepwise selection process 3 variables remained (*p* < .1): AP length (*p* = .053), intermediate T2 signal (IT2) volume (*p* = .002), and nodular border (*p* = .001). Figure 1(a)–(d) demonstrate two LMS and two DF cases with likert scores correctly and incorrectly predicting malignancy. When the model was implemented back into the training dataset it demonstrated a sensitivity of 70.7%, specificity of 76.2%, NPV of 70.3% and PPV of 76.5% (Table 5). The sensitivity was therefore below the threshold of 85%, indicating that the study would not proceed into the validation phase. The sensitivity, specificity, NPV and PPV of the radiologist suspicion score were 74.7%, 70.4%, 71.3% and 73.9% respectively (Table 6). The sensitivity analyses using a cut-off from the suspicion score of 3 for diagnosis of LMS suggested there was no improvement of overall model performance. Sensitivity improved at the expense of specificity (Sensitivity = 94.5%, Specificity = 46.9%, ROC AUC = 70.71%).

**Table 4.** Final diagnostic classification model (*n* = 174).

	β coef	95% CI	*p*-value
AP length	0.02	0.008, 0.03	0.001
IT2 volume			
1%–25%	−2.51	−3.86, −1.15	<0.001
26%–50%	−1.23	−2.49, 0.03	0.056
51%–75%	−2.07	−3.31, −0.83	0.001
76%–100% (ref)	-	-	-
Nodular border			
Yes (ref)	-	-	-
No	−1.44	−2.28, −0.60	0.001

**Table 5.** Sensitivity and specificity of the diagnostic classification model (*n* = 176).

ROC AUC = 73.4%		Model classification	
		DF	LMS
Histological subtype	DF	64	20
		**Specificity = 76.2%**	23.8%
		**NPV = 70.3%**	23.5%
	LMS	27	65
		29.3%	**Sensitivity = 70.7%**
		29.7%	**PPV = 76.5%**

**Table 6.** Sensitivity and specificity of radiologist suspicion score as a diagnostic tool (*n* = 172).

ROC AUC = 72.55%		Diagnosis from suspicious score	
		DF	LMS
Histological subtype	DF	57	24
		**Specificity = 70.4%**	29.6%
		**NPV = 71.3%**	26.1%
	LMS	23	68
		25.3%	**Sensitivity = 74.7%**
		28.7%	**PPV = 73.9%**

## Discussion

To our knowledge, this is the largest imaging study attempting to create a diagnostic model distinguishing DF from uterine LMS. Recognizing the real-world challenges, we did not include simple fibroids in the control group which are more easily distinguished from LMS. Additionally, only LMS was included in the malignant cohort. We also embedded assessment of reproducibility into our study design in order to inform the choice of imaging features to be taken forward into the model. The sensitivity of the diagnostic classification model from the training set was unfortunately, deemed too low to progress to validation.

There are several possible reasons why a diagnostic model could not be generated with sufficient sensitivity or specificity to distinguish DF from LMS. This may be due the fact that objective MRI features overlap significantly between DF and LMS. The control group may have been affected by selection bias as not all DFs undergo surgery and therefore lacked histological confirmation for study inclusion. Although intralesional haemorrhage/T1 bright components and lack of central enhancement have previously been identified as showing a stronger association with LMS^12^ we were unable to explore this due to insufficient data. From the univariate analysis, five features were not taken forward to multi-variate analysis and diagnostic modelling. These were location of mass in myometrium, feeding vessel, internal vessels, enlarged nodes, and peritoneal disease. The fact that location in the myometrium was not a predictor for sarcomatous change suggests that both DF and LMS can arise from anywhere in the myometrium. Assessing nodal involvement on MRI was based on size criteria and using morphological features of nodes (including size) on anatomical sequences has limitations. Feeding and internal vessels have been reported as features of sarcomatous change, however our analysis shows poor inter-observer agreement between the radiologists suggesting these features may be too subjective to apply to a diagnostic model.

Since completion of this study, a consensus statement for MRI evaluation of uterine masses for risk of leiomyosarcoma has been published.^22^ Unfortunately, we had insufficient data to include DWI and ADC (apparent diffusion coefficient). A further recent consensus from the International Society of Gynecologic Endoscopy also recommends contrast enhanced MRI.^23^ Contrast enhanced examinations were also lacking from our data. However, the limited size and inconsistency between some of the referenced studies is such that the relative contributions of DWI and contrast enhanced MRI have not been confirmed. For example, the number of patients with LMS in one study proposing use of DWI was 5 and another^16^ proposing contrast enhanced MRI included 10 patients with LMS.^13^ The heterogeneity in the imaging data available highlights inconsistencies in clinical practice. Ultrasound is often utilised as the first line investigation for pelvic symptoms and a recent ultrasound study has showed that both LMS and STUMP tumours showed a high percentage of circumferential and intra-lesional vascularisation which contributes to the evidence that vascularity is of relevance to the differential diagnosis.^24^ It is possible that some of the masses in this study were incidental findings not interrogated further with specialist uterine MRI. This reinforces the need for standardised MRI protocols. In our ongoing experience as a tertiary referral centre for soft tissue sarcoma, baseline imaging of patients with subsequent diagnosis of uterine LMS often lacks contrast enhanced or DW imaging as it is not suspected at the time of imaging.

Although intermediate T2 signal was confirmed to be useful in this study, neither peritoneal metastases nor malignant nodes which were included in the recommendations, were present in enough cases of LMS to be useful distinguishing features. After completion of data collection, a study has been published which retrospectively reviewed qualitative MRI features distinguishing LMS (*n* = 19) and leiomyomas (*n* = 25).^25^ This identified seven features associated with LMS; T2 hyperintensity, enhancing finger-like projections, necrosis, intratumoral haemorrhage, T2 heterogeneity, heterogeneity on post contrast T1 and an ill-defined border on post contrast T1. Using these features in an MRI predictive score, they found a score of 0–3 to have a 100% NPV for LMS and a score of 6-7 to have a 100% PPV for LMS. Our results were less promising which is likely due to inclusion only of degenerating fibroids which is a more challenging diagnostic scenario and closer to the diagnostic challenge faced by clinical radiologists.^26^

Assessment of inter-observer variability in this study provides some valuable insights which have been lacking in the literature. The size of the lesion in all dimensions had a strong kappa agreement (all >0.75). However morphological features showed only poor or moderate kappa agreement at best. This analysis suggests that morphological assessment of suspicious myometrial masses (albeit with a reporting lexicon) has significant inter-observer variability contributing to the challenge of constructing an effective objective diagnostic classification model.

Overall the diagnostic model which incorporated AP (anteroposterior) length, intermediate T2 signal and a nodular border did not confer a diagnostic advantage over the radiologists suspicion score showing similar sensitivity, specificity, NPV and PPV (70.7%, 76.5%, 70.3% and 76.5% compared with 74.7%, 70.4%, 71.3% and 73.9% respectively).

Although this MRI study has analysed the largest number of histologically proven cases of LMS:DF, it is possible that the sample size was not large enough, potentially leading to sampling error. We did not perform a formal sample size calculation, instead choosing a pragmatic approach in this rare cancer, to use all available data. We also acknowledge that stage and grade of tumours may be influential to imaging appearances but the limited size of the dataset and data missingness did not allow for sub-analysis. This study indicates that morphology alone is unlikely to be a robust discriminator between DF and LMS. Other imaging parameters such as ADC and metrics derived from contrast enhanced MRI should ideally be explored but data from standardized acquisition are unlikely to be widely available in this rare cancer. Given the lack of distinguishing MRI parameters, future work should aspire to assess combined clinic-radiological models for example by considering peri/post menopausal status, rate of growth of a mass and post menopausal bleeding.

In the absence of a robust diagnostic tool, current recommendations that laparoscopic power morcellators should not be used in women aged 50 years and over remain relevant.^25^ We would suggest that all patients with complex myometrial masses should be counselled about the risks when offered minimally invasive treatments which could increase tumour dissemination if LMS is the underlying pathology. This has been highlighted in a joint statement from the Royal College of Obstetricians and Gynecologists, Royal College of Radiologists and Sarcoma UK.^27^

## Conclusion

Morphological MRI imaging features alone are not sufficient to obviate the need for pathological confirmation prior to non-surgical management of complex uterine mass lesions. All patients with complex myometrial masses should be counselled regarding risks when offered minimally invasive interventions which could result in tumour dissemination if LMS is confirmed.

## Supplemental Material

Supplemental Material - Comparison of MRI imaging features to differentiate degenerating fibroids from uterine leiomyosarcomas Supplemental Material for Comparison of MRI imaging features to differentiate degenerating fibroids from uterine leiomyosarcomas by William W Loughborough, Andrea G Rockall, Tanja Gagliardi T, Laura Satchwell, Emily Greenlay, Piers Osborne, Nishat Bharwani, Thomas Ind, Ayoma Attygalle, Dione Lother, Georgina Hopkinson, Robin Jones, Charlotte Benson, Aisha Miah, Aslam Sohaib A, and Christina Messiou C in Rare Tumors

## Appendix

### Abbreviations

DF: Degenerating fibroids
LMS: Leiomyosarcoma
MRI: Magnetic Resonance Imaging
MR: Magnetic Resonance
LDH: Lactate dehydrogenase
T1: T1 weighted MRI sequence
T2: T2 weighted MRI sequence
DWI: Diffusion weighted imaging sequence
ADC: Apparent diffusion coefficient
NHS: National Health Service
IRAS: Integrated Research Application System
PACS: Picture archiving and communication system
CRF: Case report form
LoA: Limits of agreement
PPV: Positive Predictive Value
NPV: Negative Predictive Value
ROC: Receiver operator characteristic
SD: Standard deviation
AP: Anteroposterior
