# Supplementary material for: Comparison of MRI imaging features to differentiate degenerating fibroids from uterine leiomyosarcomas
Source: Rare Tumors. 2025 Apr 11;17:20363613251327080. doi: 10.1177/20363613251327080 (PMC12033571; doi:10.1177/20363613251327080)
Supplement: Supplemental Material - Comparison of MRI imaging features to differentiate degenerating fibroids from uterine leiomyosarcomas [file sj-pdf-1-rtu-10.1177_20363613251327080.pdf]

Radiologist initials

Patient IDOL enrolment number

Template Case Report Form

Study no:

IDOL – CCR
Template Case Report Form

Date of CRF completion
☐☐/☐☐/☐☐☐☐

Patient Details

IDOL enrolment number: ☐☐☐☐☐☐

Histological Subtype: ☐ Degenerated Fibroid ☐ Leiomyosarcoma

Any further histological information;

Source of histology: ☐ Hysterectomy ☐ Myomectomy ☐ Biopsy

Date of histological report : ☐☐/☐☐/☐☐☐☐
(dd/mm/yyyy)

If no histology, length of clinical follow up (years then months) ___________

If no histology, nature of clinical follow up (ED attendance, OP clinic etc) ___________

Date of MRI scan: ☐☐/☐☐/☐☐☐☐
(dd/mm/yyyy):

Patient age ___________

LDH avaliable at time of initial MRI Yes☐ No☐

If available, LDH at diagnosis: ___________IU/I

Cohort: ☐ Training ☐ Validation

Radiologist initials

Patient IDOL enrolment number

Name of reader Signature

Date of CRF completion

☐☐/☐☐/☐☐☐☐

MR report

**Imaging feature of concerning lesion**	
Craniocaudal length	**mm**
AP length	**mm**
Transverse length	**mm**
>1 fibroid?	☐ Yes ☐ No
If > 1 fibroid, do the additional fibroids fit MRI inclusion criteria? If yes, complete a form for each.	☐ Yes ☐ No
1 or greater homogenously low T2 signal fibroid	☐ Yes ☐ No
Location of mass in myometrium	Submucosal ☐ Intramural ☐<br>Subserosal ☐ Other __________
**Characterization of concerning lesion**	
Focal protrusion into myometrium	☐ Yes ☐ No
Nodular border	☐ Yes ☐ No
Adjacent organ invasion	☐ Yes ☐ No
Feeding vessel	☐ Yes ☐ No
Internal vessels	☐ Yes ☐ No
**Intermediate T2 signal**	
Solitary area	☐ Yes ☐ No<br>☐ Heterogenous ☐ Homogenous<br>☐ N/A
Multifocal areas	☐ Yes ☐ No<br>☐ Heterogenous ☐ Homogenous<br>☐ N/A
Specify subjective volume of intermediate T2 signal within the mass	☐ 1-25% ☐ 26-50%☐ 51-75% ☐<br>100% ☐N/A
**Low T2 signal**	
Solitary area	☐ Yes ☐ No<br>☐ Heterogenous ☐ Homogenous<br>☐ N/A
Multifocal areas	☐ Yes ☐ No<br>☐ Heterogenous ☐ Homogenous<br>☐ N/A
Specify subjective volume of low T2	☐ 1-25% ☐ 26-50%☐ 51-75% ☐

Radiologist initials	Patient IDOL enrolment number

signal within the mass	100% ☐N/A
**Necrosis**	
Solitary area	☐ Yes ☐ No
Multifocal areas	☐ Yes ☐ No
Specify subjective volume of necrotic signal within mass	☐ 1-25% ☐ 26-50%☐ 51-75% ☐ 100% ☐N/A
**High T1**	
High T1	☐Hemorrhage/protein ☐Fat ☐Uncertain
Solitary area	☐ Yes ☐ No ☐N/A
Multifocal areas	☐ Yes ☐ No ☐N/A
Specify subjective volume of high T1 within the mass	☐ 1-25% ☐ 26-50%☐ 51-75% ☐ 100% ☐N/A
Rim of high T1 surrounding mass?	☐ Yes ☐ No ☐N/A
**Restricted diffusion**	
Restricted diffusion?	☐ Yes ☐ No ☐N/A
Mean ADC	Value =
ROI area for measured ADC	Value =
Specify subjective volume of restricted diffusion within the mass	☐ 1-25% ☐ 26-50%☐ 51-75% ☐ 76-100% ☐N/A
**Fibrosis**	
Fibrotic signal on DWI	☐ Yes ☐ No
Specify subjective volume of fibrosis within the mass	☐ 1-25% ☐ 26-50%☐ 51-75% ☐ 76-100% ☐N/A
**Enhancement**	
Enhancement	☐ None ☐ Low ☐ High
If enhancement, heterogeneous or homogenous?	☐ Heterogeneous ☐ Homogenous ☐N/A
If enhancement, are there areas of non-enhancement?	☐ Central ☐ Non central ☐No enhancement ☐N/A
If contrast enhanced sequence(s), is there a dynamic constrast enhanced (DCE) sequences?	☐ Yes ☐ No
If DCE – is there early enhancement	☐ Yes ☐ No ☐N/A
If DCE – is there late enhancement	☐ Yes ☐ No ☐N/A
Enlarged nodes	☐ Yes ☐ No
Peritoneal disease	☐ Yes ☐ No
Ascites	☐ Yes ☐ No
Adequate images	☐ Yes ☐ No
Subjective Likert score of likelihood of malignancy	☐ 1 – very unlikely<br>☐ 2 - unlikely

Radiologist initials		Patient IDOL enrolment number

	☐ 3 - equivocal<br>☐ 4 – likely<br>☐ 5 - very likely.
Notes	

Radiologist initials	Patient IDOL enrolment number

Lexicon for imaging features

Imaging feature		Definition
Measurements definitions		These measurements refer to the lesion in question (rather than the uterus etc)<br><br>Longest measurement in following planes;<br>AP and transverse – axial sequence<br>Craniocaudal – sagittal sequence. If no sagittal then coronal sequence.
MRI inclusion criteria		> 20% high T2 signal and/or two or more areas of intermediate T2 signal
Myometrial protrusion		Focal protrusions of the suspicious myometrial mass into the myometrium
Nodular border	Lakhman et al[1]	Is the border smooth or nodular (either focally or diffusely)?
Organ invasion		Unequivocal extension of myometrial tumor signal into adjacent organ(s)
Vessel (either feeding vessel(s) or intra-tumoral vessel(s))	Lakhman et al[1]	Unequivocal round and/or serpiginous flow voids on T2 weighted sequence(s).
Intermediate T2 signal	Sadowski et al[2]	Higher signal than iliopsoas, lower signal than CSF. Similar intensity to outer myometrium
Low T2 signal	Sadowski et al[2]	Lower signal than iliopsoas
Necrotic/cystic mass	Lakhman et al[1]<br>Sahdev et al[3]<br>Tanaka et al[4]	Lower T1 signal than iliopsoas<br>High T2 (equal to or higher than CSF and/or urine).
High T1;Haemorrhage/protein<br>Fat<br>Uncertain	Sadowski et al[2]<br>Tanaka et al[4]	T1 signal equal to or higher than fat<br>High T1 not suppressing on fat sat<br>High T1 suppressing on fat sat<br>No fat saturated sequence
Restricted diffusion	Sadowski et al[2]	B-value signal higher than CSF<br>ADC signal lower than outer myometrium
ADC	Sato et al[5]	Largest ROI area measured for ADC value in solid areas of myometrial mass but not in cystic or hemorrhagic areas (as defined above)
Fibrotic signal	Sadowski et al[2]	Low signal on T2, b value and ADC sequences (dark, dark)
Enhancement		None (no enhancement between pre and post contrast).<br>Low – iso-enhancement to skeletal muscle.<br>High – equal or greater than myometrium
Early enhancement		30s – 60s
Late enhancement		>60s
Homogenous or heterogeneous enhancement	Lakhman et al[1]	Is enhancement homogenous throughout mass or heterogeneous.
Nodes	Lakhman et al[1]	Nodes > 8mm.
Ascites	Sahdev et al[3]	Free fluid in the Pouch of Douglas or elsewhere
Peritoneal disease		Peritoneal nodules which enhance and/or restrict.

Radiologist initials	Patient IDOL enrolment number

1. Lakhman Y, Veeraraghavan H, Chaim J, Feier D, Goldman DA, Moskowitz CS, Nougaret S, Sosa RE, Vargas HA, Soslow RA, Abu-Rustum NR. Differentiation of uterine leiomyosarcoma from atypical leiomyoma: diagnostic accuracy of qualitative MR imaging features and feasibility of texture analysis. European radiology. 2017 Jul 1;27(7):2903-15.
2. Sadowski EA, Thomassin-Naggara I, Rockall A, Maturen KE, Forstner R, Jha P, Nougaret S, Siegelman ES, Reinhold C. O-RADS MRI risk stratification system: guide for assessing adnexal lesions from the ACR O-RADS Committee. Radiology. 2022 Apr;303(1):35-47.
3. Sahdev A, Sohaib SA, Jacobs I, Shepherd JH, Oram DH, Reznek RH. MR imaging of uterine sarcomas. Am J Roentgenol 2001;177(6):1307–1311.
4. Tanaka YO, Nishida M, Tsunoda H, Okamoto Y, Yoshikawa H. Smooth muscle tumors of uncertain malignant potential and leiomyosarcomas of the uterus: MR findings. Journal of Magnetic Resonance Imaging: An Official Journal of the International Society for Magnetic Resonance in Medicine. 2004 Dec;20(6):998-1007.
5. Sato K, Yuasa N, Fujita M, Fukushima Y. Clinical application of diffusion-weighted imaging for preoperative differentiation between uterine leiomyoma and leiomyosarcoma. Am J Obstet Gynecol 2014;210(4).
